# In Vivo Evaluation of the Mechanical Strength of a Slide Lengthening Technique With a Locking Mechanism Using a Rabbit Model

**DOI:** 10.7759/cureus.12387

**Published:** 2020-12-30

**Authors:** Dai Iwase, Kentaro Uchida, Yukie Metoki, Hiroyuki Sekiguchi, Jun Aikawa, Takashi Matsuo, Atsushi Matsuo, Masashi Takaso

**Affiliations:** 1 Department of Orthopaedic Surgery, Kitasato University, School of Medicine, Sagamihara, JPN; 2 Medical Sciences Research Institute, Shonan University, Chigasaki, JPN; 3 Department of Orthopaedic Surgery, Minamitama Othopaedic Hospital, Kanagawa, JPN; 4 Department of Orthopaedic Surgery, Saga Handicapped Children’s Hospital, Kinryu, JPN

**Keywords:** tendon, sliding lengthening, z-lengthening

## Abstract

Background

There are many reports of Achilles tendon lengthening procedures for equinus deformity of the ankle. We previously modified an Achilles tendon lengthening to prevent overextension with a locking mechanism suture before performing a sliding lengthening. The purpose of this study was to compare the biomechanical properties of the locking mechanism suture with sliding lengthening (L-SL) and Z-lengthening (ZL) using a rabbit model.

Methods

Thirty-six male Japanese white rabbits were assigned to two groups - half undergoing the L-SL technique and half undergoing the ZL technique on the flexor hallucis longus (FHL) tendon. Six rabbits in each group were sacrificed at one week, three weeks, and six weeks postoperatively and evaluated, while five rabbits underwent radiographical and biomechanical evaluation and one underwent histological evaluation.

Results

In extension length, L-SL was significantly lower than ZL one week postoperatively. In the L-SL group, elongation one week postoperatively was significantly lower than that three and six weeks postoperatively. In the ultimate failure load, L-SL was significantly higher than ZL one and three weeks after lengthening. In the L-SL group, the ultimate failure load one week postoperatively was significantly lower than that three and six weeks postoperatively. In the ZL group, there were significant differences at all time points.

Conclusion

L-SL had higher mechanical property in vivo.

## Introduction

Equinus deformity of the ankle is one of the most common orthopedic problems in children with spastic cerebral palsy [1]. A previous study on the prevalence of certain gait patterns in children with cerebral palsy indicated that 64% of children with spastic hemiplegia have an equinus deformity [2]. Equinus disrupts the gait cycle by decreasing stability in the stance phase and causing inadequate clearance in the swing phase [3]. Optimal operative or nonoperative treatment is needed for the equinus of the ankle in patients with cerebral palsy.

Achilles tendon lengthening is one of the most commonly used orthopedic procedures to improve equinus deformity. Although many different lengthening procedures have been described [4, 5], two of the most common are the Z-lengthening technique (ZL) and sliding lengthening technique (SL). These methods are often used for fixed contractures [6, 7].

After an operation, the immobilization period varies from three to seven weeks to ensure healing before returning to full function or exercise [8]. Blaiser and White have recommended three weeks of immobilization after percutaneous sliding heel-cord lengthening [6], whereas Renshaw et al. have recommended six weeks in a short leg cast [9]. Some authors have suggested four weeks or more of short leg cast immobilization after ZL [10]. However, prolonged immobilization following operative treatment results in muscle weakness, joint contracture, slow recovery, and rehabilitation. Treatment of equinus deformity, therefore, requires lengthening techniques with a reduced immobilization period [5].

To achieve early mobilization, several lengthening methods having higher mechanical properties have been developed [5, 11]. Hashimoto et al. previously showed that the SL with mattress sutures technique has higher ultimate tensile strength than ZL for flexor pollicis longus tendons, flexor digitorum superficialis tendons, and flexor digitorum profundus tendons of fresh cadavers in vitro [12]. We previously modified White’s SL [13] to allow extension by an exact amount and prevent overextension with a locking mechanism suture with sliding lengthening (L-SL) before performing extension [14-17]. However, the efficacy of L-SL in vivo remains to be determined.

The purpose of this study was to compare the biomechanical properties of L-SL and ZL using a rabbit model in vivo to assess their utility in allowing earlier mobility.

## Materials and methods

The experimental protocol was approved by the Kitasato University School of Medicine Animal Care Committee (reference number: 2019-133). Thirty-six male Japanese white rabbits with an average weight of 3-3.5kg were purchased from Oriental Yeast Co., Ltd (Tokyo, Japan). We randomly divided them into two groups: L-SL and ZL groups (each n=18). We operated on the right flexor hallucis longus tendon (FHL) of each rabbit under anesthesia consisting of medetomidine hydrochloride (ZENOAQ, Fukushima, Japan), butorphanol tartrate (Meiji Seika Pharma, Tokyo, Japan), and midazolam (Sandoz, Tokyo, Japan) at a ratio of 3:1:1.

**Figure 1:**
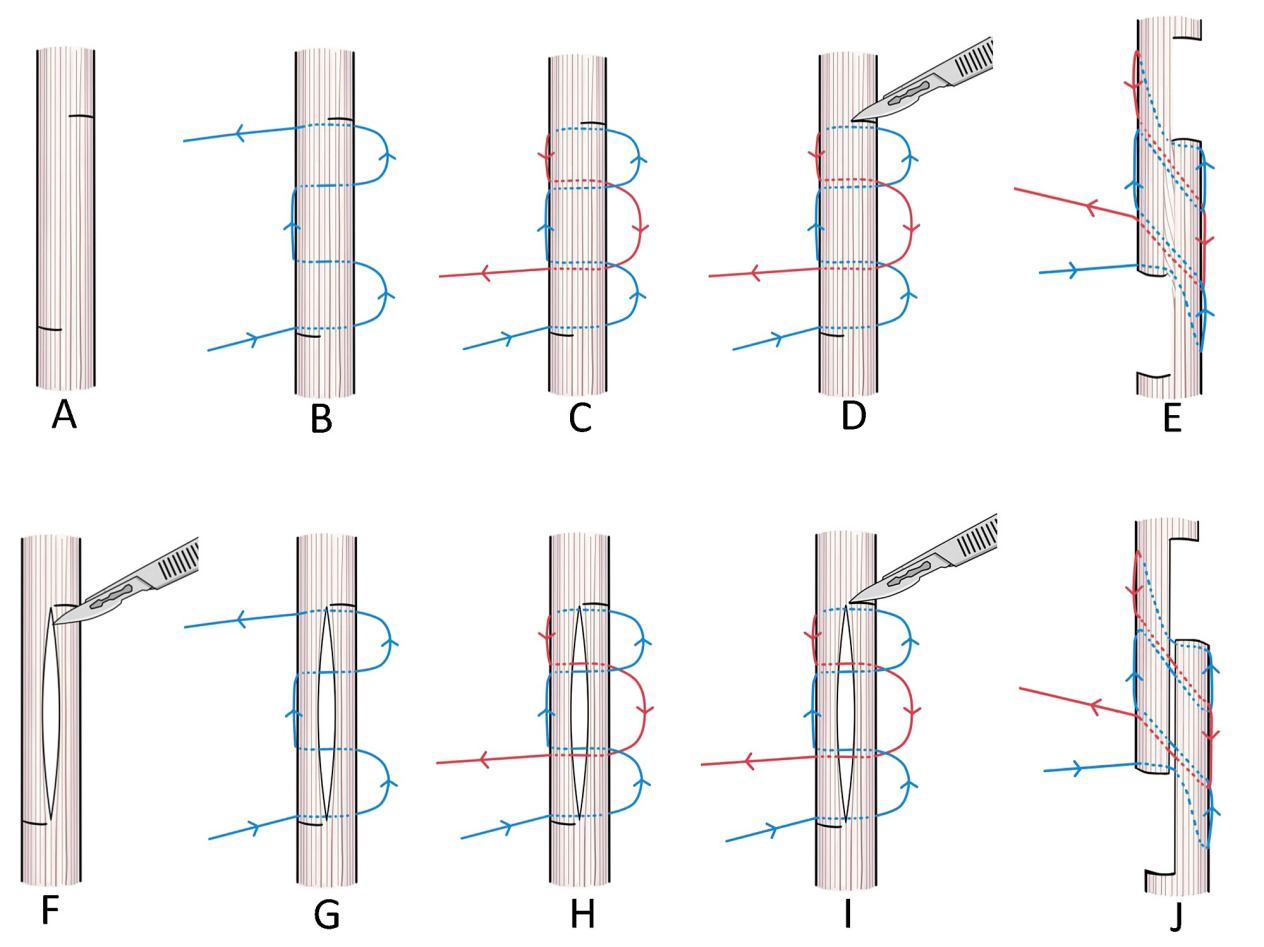
The L-SL (upper) and ZL (lower) methods of tendon lengthening L-SL - locking mechanism suture with sliding lengthening; ZL - Z-lengthening

For L-SL, we first marked a length of 15 mm (Figure 1A) with 4-0 polypropylene as in Figure 1B-C. Each loop length was 10 mm, which was equivalent to the planned extension amount (Figure 1C). Next, we made symmetrical transverse half incisions on the 15 mm mark (Figure 1D) and carefully extended the tendon (Figure 1E). Finally, a stainless steel soft wire was applied to both ends of the incision. For ZL, we first marked the tendon in a Z-shape and then performed a vertical incision (Figure 1F). After that, suturing was done the same way as for L-SL (Figure 1G-H). Next, we made symmetrical transverse half incisions on the markings (Figure 1I) and extended the tendon (Figure 1J).

Finally, two stainless steel soft wires were positioned proximally and distally to allow elongation to be evaluated radiologically. No immobilization was applied, and the animals were kept in a cage. Six rabbits each were sacrificed at one, three, and six weeks postoperatively and evaluated (n=6 in each group for respective time points). Five rabbits were used for radiographical and biomechanical evaluation. The remaining rabbit was used for histological evaluation.

Radiographical evaluation

**Figure 2:**
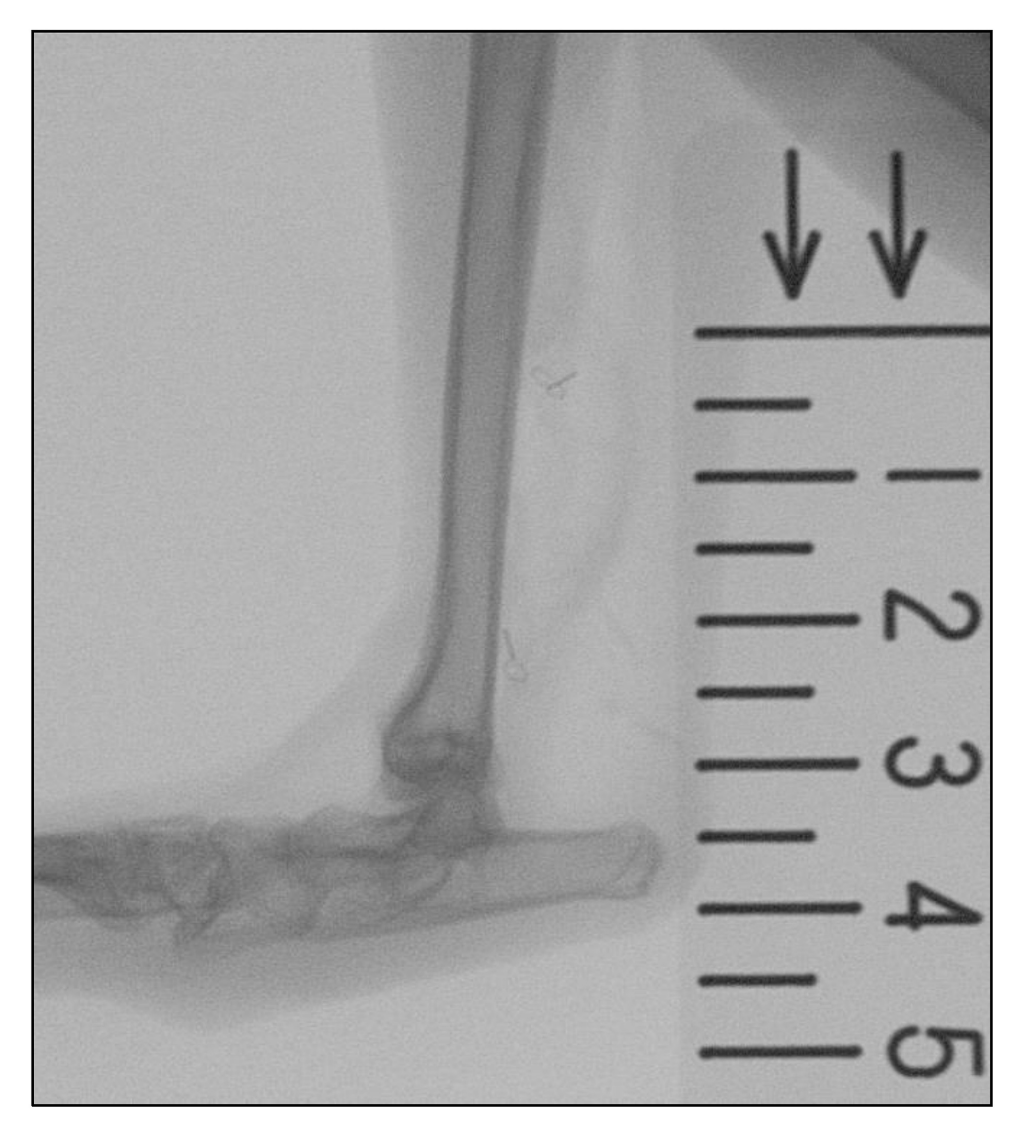
Radiograph of a treated ankle with radiopaque wires marking the ends of the incision

To measure the amount of extension after tendon lengthening, lateral radiographs of the leg were taken immediately after surgery and one, three, and six weeks postoperatively under anesthesia. Extension length was measured between the proximal and distal wires (Figure 2).

Biomechanical evaluation

**Figure 3:**
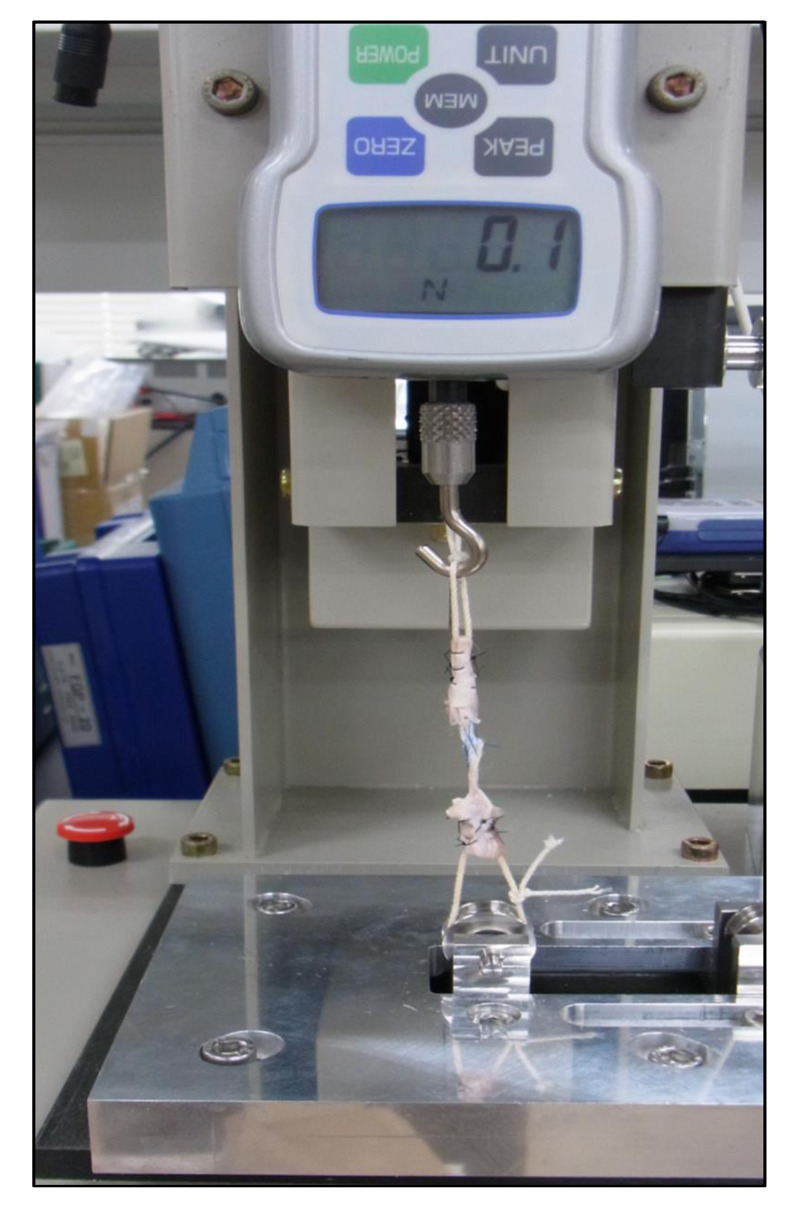
Use of a digital force gauge to conduct traction experiments at 20 mm/min until failure

A digital force gauge FGPX-20 (Nidec-Shimpo Corp., Kyoto, Japan) was used for this study. Following sacrifice, we conducted traction experiments at 20 mm/min until failure and then calculated the ultimate failure load from the load-displacement curve (Figure 3).

Histology

The tendon specimens were fixed in 4% paraformaldehyde solution for 48 hours at 4°C. They were then immersed in 5% carboxymethyl cellulose (CMC) gel for 10 min, transferred to hexane, and completely frozen using solid carbon dioxide. The tendon specimens were then cut into 6-μm-thick sections with a tungsten carbide blade. The sections were dried at -25°C and stained with hematoxylin and eosin (Kureha Special Laboratory Co., Ltd., Tokyo, Japan).

Statistical analysis

We used Student’s t-tests to compare differences between SL and ZL at each postoperative interval after sacrifice. The Kolmogorov-Smirnov test was used to determine whether or not the data were normally distributed. The Tukey-Kramer method was used to compare differences in the postoperative period after the sacrifice in each group. All statistical analyses were performed with SPSS software (Version 25.0; IBM Inc., Armonk, USA). A p-value of < 0.05 was considered statistically significant.

## Results

Radiographical evaluation

The distance between the proximal and distal wires of the L-SL group was significantly lower than that of the ZL group one week postoperatively (p=0.048, Table 1). In the L-SL group, elongation at one week postoperatively was significantly lower than that at three and six weeks (p=0.047 and p=0.043, respectively). On the other hand, there was no significant difference in the ZL group among all periods (p=0.050).

**Table 1:** Extension length following tendon lengthening ^a^p<0.05, L-SL versus ZL by student T-test. ^b^p<0.05, one week versus three and six weeks by Tukey-Kramer method. L-SL - locking mechanism suture with sliding lengthening; ZL - Z-lengthening

	One week	Three weeks	Six weeks
L-SL	25.8±8.5	84.0±22.9^b^	85.2±10.2^b^
ZL	60.0±11.9^a^	100.2±14.6	98.6±6.8

Biomechanical evaluation

Although the ultimate failure load of the L-SL group was significantly higher than that of the ZL one and three weeks after lengthening (p=0.001 and p=0.028, respectively, Table 2), that of both groups increased with time. In the L-SL group, the ultimate failure load at one week postoperatively was significantly lower than that at three and six weeks (p<0.001 and p=0.028, respectively). In the ZL group, there were significant differences at all time points (p<0.001).

**Table 2:** Ultimate failure load ^a^p<0.05, L-SL versus ZL by student T-test. ^b^p<0.05, one versus three and six weeks by Tukey-Kramer method. ^c^p<0.05, three versus six weeks by Tukey-Kramer method. L-SL - locking mechanism suture with sliding lengthening; ZL - Z-lengthening

	One week	Three weeks	Six weeks
L-SL	7.8±1.2	42.4±2.0^b^	60.1±8.1^b^
ZL	1.0±0.0^a^	35.6±1.5^a,b^	56.4±3.0^b,c^

Histology

**Figure 4:**
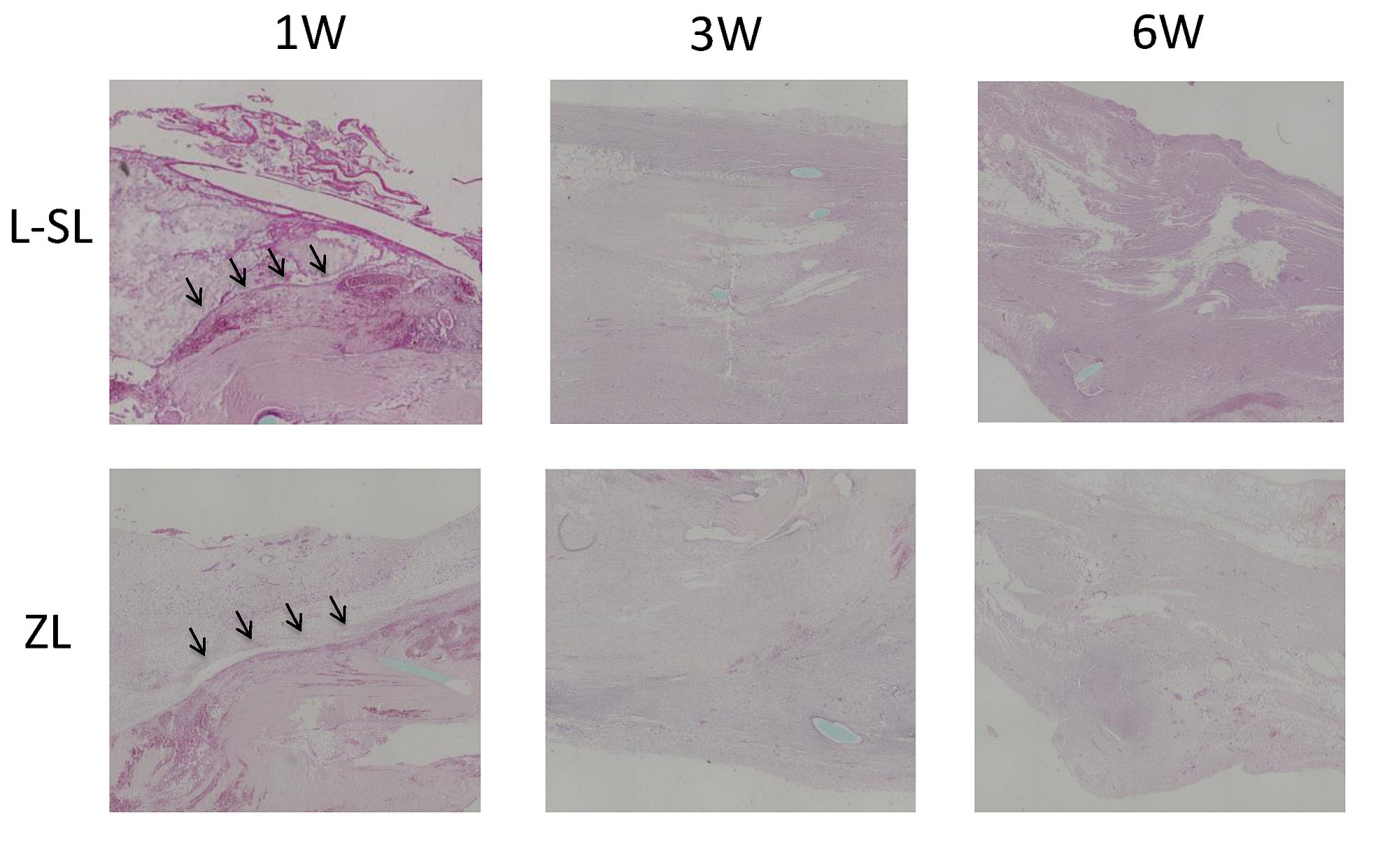
Histological sections at one, three, and six weeks showing early one-week fiber continuity with L-SL (arrows, first row) L-SL - locking mechanism suture with sliding lengthening; ZL - Z-lengthening; W - week

In the ZL group, fiber continuity was not confirmed at one week but was confirmed in the L-SL group (Figure 4). Continuity was confirmed in both the SL and ZL groups at three and six weeks postoperatively.

## Discussion

Previous studies have suggested that earlier mobilization after tendon lengthening can prevent adhesions and contracture, as a result, lead to quicker recovery [5, 17]. However, earlier mobilization may also cause calcaneal gait with overlengthening [18]. To improve these issues we have investigated L-SL. In this study, L-SL had higher mechanical properties than ZL one week and three weeks postoperatively in vivo rabbit model. In addition, continuity of tendon tissues was observed in L-SL one week after the operation. These results suggest that L-SL may have higher mechanical properties due to the maintenance of continuity in the early postoperative period. L-SL may allow earlier mobilization than ZL in the clinical setting.

Overlengthening frequently occurs after Achilles tendon lengthening [19, 20]. Overlengthening of the Achilles tendon causes crouched or calcaneus gait. In our study, we evaluated whether L-SL prevented overlengthening. L-SL resulted in significantly less elongation than ZL one week after the operation. L-SL may help to sustain the tendon length after lengthening, albeit its effect was examined over only a short period. 

Several limitations of this study warrant mention. First, the results obtained from small animal models are not always relevant for human applications. Additional studies using large animal models are therefore important, and essential to confirm our present findings. Second, regarding the animals’ lack of immobilization after tendon lengthening, overlengthening could occur via animal ambulation. Further investigation using an animal model with fixation to immobilize the leg is needed.

## Conclusions

In conclusion, L-SL had higher mechanical property in vivo. This property may allow early active exercise in clinical settings.
